# Liquid Mixing Based on Electrokinetic Vortices Generated in a T-Type Microchannel

**DOI:** 10.3390/mi12020130

**Published:** 2021-01-26

**Authors:** Chengfa Wang

**Affiliations:** Department of Marine Engineering, Dalian Maritime University, No.1 Linghai Road, Dalian 116026, China; wangcf08@dlmu.edu.cn

**Keywords:** micromixer, electrokinetic vortices, T-type microchannel, zeta potential ratio, length ratio

## Abstract

This article proposes a micromixer based on the vortices generated in a T-type microchannel with nonuniform but same polarity zeta potentials under a direct current (DC) electric field. The downstream section (modified section) of the outlet channel was designed with a smaller zeta potential than others (unmodified section). When a DC electric field is applied in the microchannel, the electrokinetic vortices will form under certain conditions and hence mix the solution. The numerical results show that the mixing performance is better when the channel width and the zeta potential ratio of the modified section to the unmodified section are smaller. Besides, the electrokinetic vortices formed in the microchannel are stronger under a larger length ratio of the modified section to the unmodified section of the outlet channel, and correspondingly, the mixing performance is better. The micromixer presented in the paper is quite simple in structure and has good potential applications in microfluidic devices.

## 1. Introduction

Microfluidic devices have been widely used in biological or chemical analysis because of the significant advantages of easy operation, low cost, and less sample consumption [1]. Since the liquid is usually laminar in microchannels due to the small Reynolds number, the effects of diffusion and convection are quite weak and the liquid mixing is a challenge in microchannels. Therefore, micromixers are the key components of microfluidic devices and have been studied extensively.

The reported micromixers are usually divided into two categories: Active micromixers and passive micromixers. Active micromixers utilize external energy sources such as electric field [2,3,4] magnetic field [5,6,7], and acoustic field [8,9,10] applied in microchannels to improve the solution mixing performance. The channel structures of these micromixers are relatively simple. By contrast, external energy sources are not required for passive micromixers, but this type of micromixer generally needs to have a complicated channel structure [11,12,13,14,15,16,17,18,19] or set obstacles [20,21,22,23] in the microchannel to disturb the laminar flow for solution mixing under a relatively high pressure generated by a syringe pump. The mixing efficiency of passive micromixers is high, but the sealing of a microfluidic chip under high pressure is a challenge. The details can be found in the review articles [24,25].

It is generally known that when a charged solid surface is in contact with an aqueous solution, the surface electrostatic charges will attract the counter-ions in the solution and then the electric double layer (EDL) forms on the surface [26]. The zeta potential is an important parameter to evaluate the surface charge density. Once a DC electric field is applied along the surface, the electroosmotic flow (EOF) will form in the EDL and drive the liquid near the surface to flow. If the channel walls have different polarity zeta potentials, electrokinetic vortices can be easily generated due to the different directions of electroosmotic flows formed on the walls. Vortices can stir the liquid, thereby enhancing the effects of diffusion and convection, so the electrokinetic vortex is an effective method to solve the mixing problem in microfluidic devices and can be used to design different types of electrokinetically-driven active micromixer. It is widely known that a metal object immersed in an aqueous solution will be induced by an external electric field. As a result, one side of the metal object obtains positive charges and another side obtains equal amounts of negative charges. The details can be found in the reference [27]. Therefore, the electrokinetic vortex can be easily formed by the induced charge electroosmotic flow (ICEO) generated on a metal object surface [27,28,29,30]. Based on electrokinetic vortices generated by ICEO, Wu and Li [31,32] designed a micromixer and conducted a systematically numerical and experimental investigation. The advantage of this kind of micromixer is that the inhomogeneous zeta potential surfaces can be obtained directly without surface modification. However, under a high electric field strength, the “Bipolar Electrochemistry” phenomenon [33] generated on conducting surfaces is unfavorable to the vortex generation, thereby affecting the mixing performance.

A metal surface can obtain different polarity zeta potentials without surface treatment. However, a non-conductive surface usually has a uniform zeta potential, so surface modification technology should be used to make the wall obtain inhomogeneous zeta potentials, then electrokinetic vortices can be formed in the microchannel. Hau et al. [34] and Stroock et al. [35] experimentally investigated the electroosmotic flow formed on a surface with different polarity zeta potentials and demonstrated that electrokinetic vortices can form near this kind of surface. Erickson and Li [36,37,38] carried out numerical studies on the electrokinetic vortices formed in a straight microchannel by designing various patterns on the microchannel walls with different polarity zeta potentials and found the degree of pattern heterogeneity greatly affect the electrokinetic vortex generation. However, it is not easy to manufacture the surface with different polarity zeta potentials since a surface in the same aqueous solution usually has the same polarity zeta potentials [39].

As described, electrokinetic vortices can easily form on a surface with different polarity zeta potentials. If the surface has different value but same polarity zeta potentials, can electrokinetic vortices still form? Recently published studies show that electrokinetic vortices can also form on such surfaces under certain conditions [40,41]. Song et al. [41] utilized polybrene (PB) coating technology to design a two-section straight microchannel with two same polarity zeta potentials (one section was modified with a smaller absolute value of negative zeta potential, and another section was not modified). and studied the evolution of the flow field in the microchannel. The results show that electrokinetic vortices can form in such a microchannel when the length ratio of the modified section to the unmodified section is large enough.

Active micromixers based on electrokinetic vortices have the advantages of easy operation and sample structure. Biddiss et al. [42] carried out an investigation on the liquid mixing performance near a non-conductive surface with different value and different polarity zeta potentials. However, so far very few published works have presented the liquid mixing based on electrokinetic vortices generated on a surface with different value but same polarity zeta potentials. In this paper, a T-type micromixer was designed utilizing electrokinetic vortices formed in the microchannel with different value but same polarity zeta potentials. The dependencies of surface zeta potentials, channel width, and other factors on the mixing performance of the micromixer were numerically investigated. The advantage of the presented micromixer is that the microchannel structure and the work system are quite simple. The micromixer only needs a small battery to work, which is conducive to the integration and miniaturization of microfluidic devices. This work provides a certain theoretical basis for practical application in the future.

## 2. Structure and Working Principle of the Micromixer

**Figure 1:**
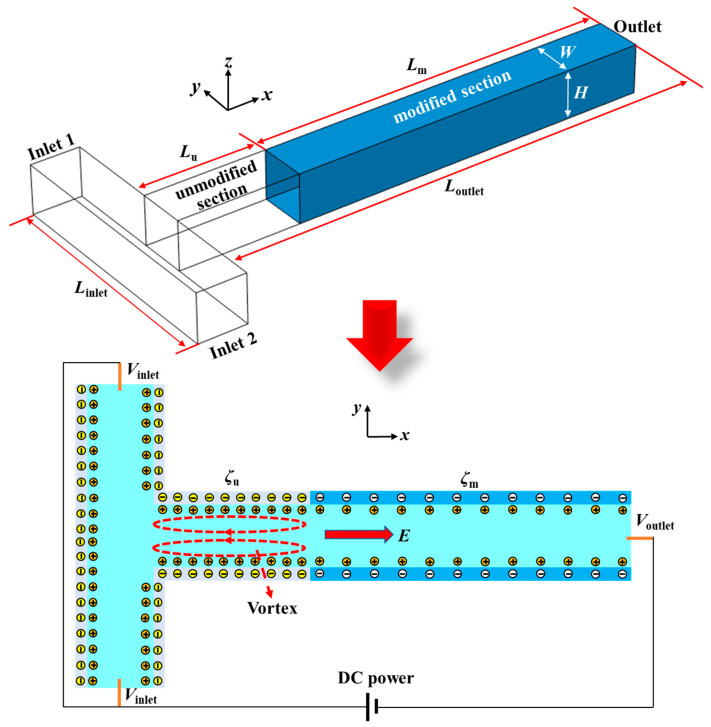
Schematic diagram of the micromixer structure and working principle. The zeta potentials of the unmodified section wall (*ζ*_u_) and the modified section (*ζ*_m_) wall are all negative, but the absolute value of *ζ*_m_ is smaller than that of *ζ*_u_.

Figure 1 displays the structure and working principle of the proposed micromixer. The structure of the micromixer is a T-type microchannel with one outlet and two inlets. The lengths of the inlet and outlet channels are *L*_inlet_ and *L*_outlet_, and the width and height of the channel are equal, namely, *W* = *H*. In this study, the dimensionless lengths of the inlet and outlet channels were set to be 10 and 30, respectively, and the characteristic length is *L*_ref_ = 10 μm. The outlet channel is divided into an unmodified section and a modified section. The lengths of the unmodified section and the modified section are *L*_u_ and *L*_m_, and the parameter of *β* = *L*_m_/*L*_u_ is defined as the length ratio of the modified section to the unmodified section. Two different DC voltages of *V*_inlet_ and *V*_outlet_ are applied at the two inlets and the outlet of the microchannel. The walls of the inlet channel and the unmodified section of the outlet channel have the same negative zeta potential (*ζ*_u_), and the negative zeta potential (*ζ*_m_) of the modified section is designed with a smaller absolute value. The parameter of *α* = *ζ*_m_/*ζ*_u_ is defined as the zeta potential ratio of the modified section to the unmodified section.

Once the charged channel walls are in contact with an aqueous solution, electric double layers (EDL) will form along the walls. When the voltages of *V*_inlet_ and *V*_outlet_ are applied, the electroosmotic flow (EOF) will be generated in the microchannels. Under the same electric field, the velocity of EOF is determined by the surface zeta potential. The larger the absolute value of surface zeta potential is, the higher the velocity of EOF is. Since the absolute value of *ζ*_m_ is smaller than that of *ζ*_u_, the velocity of EOF formed in the modified section is smaller than that in the unmodified section. Therefore, the liquid in the modified section will impede the flow of liquid in the unmodified section. When the EOF velocity difference of the unmodified section to the modified section is large enough, the “impede effect” of the modified section liquid will become strong. As a result, the liquid in the unmodified section will flow back, hence the electrokinetic vortex generates in the microchannel, as shown in Figure 1. The formed electrokinetic vortices stir the laminar flow in the microchannel and then realize liquid mixing. The parameters of the zeta potential ratio (*α*) and the length ratio (*β*) of the modified section to the unmodified section have an important influence on the “impede effect” of modified section liquid, thereby affecting the strength of electrokinetic vortices and the corresponding mixing performance.

## 3. Theoretical Model

After a DC electric field is applied in the microchannels, electrokinetic vortices will form, thereby mixing the solution. Therefore, this model contains an electric field, a flow field, and a concentration field.

### 3.1. Electric Field

The DC electric potential (*V*) field in the microchannels is described by Laplace’s equation:

(1) $$\nabla^{2}V=0$$

The relationship between the electric potential and the electric field strength (***E***) is as follows:

(2) E=−∇V

Since all the channel walls are electrically insulated, the boundary condition is

(3) n⋅E=0

where ***n*** is the unit normal vector on the boundary walls.

The voltages of *V*_inlet_ and *V*_outlet_ are applied at two inlet boundaries and one outlet boundary, respectively, and the boundary conditions are

(4) $$V=V_{\mathrm{inlet}}$$

(5) $$V=V_{\mathrm{outlet}}$$

In this work, the outlet boundary was grounded, namely, *V*_outlet_ = 0. The electric field strength (***E***) in the outlet channel is set as a constant value of 50 V/cm by adjusting the inlet voltage of *V*_inlet_.

### 3.2. Flow Field

The Navier–Stokes equation and the continuity equation govern the distribution of the flow field in the microchannels. Their expressions in steady-state are as follows:

(6) $$\rho U\cdot \nabla U=-\nabla p+\mu \nabla^{2}U$$

(7) ∇⋅U=0

where *ρ* = 1000 kg/m^3^ is the solution density, ***U*** is the velocity vector, *μ* = 0.001 Pa·s is the dynamic viscosity of the solution, and *p* is the pressure.

In this study, the flow of the solution in the microchannels is dependent on the EOFs formed on the charged walls by the DC electric field, and there is no pressure-driven flow. Thus, the boundary conditions of the inlets and outlet are

(8) p=0

In this model, the net charge density is zero except in EDLs, but compared with the scale of the microchannel, the EDL thickness is negligible. Thus, this model does not consider the body force acting in the EDL by the DC electric field and the distribution of the flow field in the EDL. Instead, a slip boundary condition (Helmholtz–Smoluchowski velocity [26,43]) was adopted to reflect the EOFs generated on the channel walls. Thus, the boundary conditions of the flow field on the channel walls are as follows:

(9) $$U=-\frac{\varepsilon_{0}\varepsilon \zeta_{m}}{\mu }E$$

on the channel walls of the modified section;

(10) $$U=-\frac{\varepsilon_{0}\varepsilon \zeta_{u}}{\mu }E$$

on the other channel walls, where *ε*_0_ = 8.85 × 10^−12^ F/m is the vacuum permittivity, ε = 80 is the relative dielectric constant of solution.

Considering that polydimethylsiloxane (PDMS) is a widely-used material in the fabrication of microchannels and its zeta potential can be controlled in the range of −1.7 mV and −57 mV by surface treatment [44], this model took *ζ*_U_ = −50 mV as a characteristic value of the zeta potential on the unmodified section walls.

The Reynolds number in this study is less than 0.1 due to the small microchannel size and the electric field intensity. With the increase of the electric field, the velocity of EOFs and the corresponding Reynolds number will increase. The electric field and the micromixer size can be adjusted according to the actual needs. However, it should be noted that the Joule heating effect from a strong electric field will play a negative effect on the biological cells in the solutions. Thus, a small electric field is used in this work.

### 3.3. Concentration Field

Assuming that the concentration is not affected by any reactions and there is no migration of ionic species, the convection-diffusion equation, which describes the concentration of the solute in water in a steady-state, is as below [45]:

(11) $$\nabla \cdot \left(-D\nabla c\right)+U\cdot \nabla c=0$$

where *c* is the solute concentration, *D* = 10^−11^ m^2^/s is the diffusion coefficient of the solute.

Since all channel walls are insulated for the solute, no flux condition (Equation (12)) was applied to the walls.

(12) $$-n\cdot \left(-D\nabla c+Uc\right)=0$$

At Inlet 1 (see Figure 1) the solute concentration is *c*_0_ = 1 mol/m^3^; at Inlet 2 the concentration is zero, that is

(13) $$c=c_{0}$$

at Inlet 1;

(14) c=0

at Inlet 2.

The solute is transported out of the outlet boundary by liquid motion, so convection dominates the solute transport and the diffusive transport can be ignored. Thus, the boundary condition at Outlet is

(15) n⋅D∇c=0

To evaluate the mixing performance of the micromixer, a mixing index is defined as follows:

(16) $$\gamma =\frac{c_{\min}}{c_{\max}}$$

where *c*_max_ and *c*_min_ are the maximum value and the minimum value of the solute concentration at the Outlet (see Figure 1). The value of *γ* is in the range of 0 to 1. It is obvious that the larger the value of *γ*, the better the mixing performance.

COMSOL MULTIPHYSICS 5.2a ^®^ was chosen to calculate the above 3D numerical model in this work. The electric field, flow field, and concentration field in the model are mutually coupled and solved simultaneously by the software. The simulation process is as follows. According to the above-mentioned governing equations, the appropriate physical fields are firstly chosen from the “Physics” module of the software. Secondly, the computational domain is drawn via the “Geometry” module. Thirdly, the boundary conditions are set in the software according to the theoretical model. Finally, the mesh is built and the model is calculated.

Mesh quality greatly affects the accuracy of the numerical result. To avoid this problem, mesh independence was examined by calculating the mixing index of *γ* under different mesh numbers. The result shows that the rate of change in *γ* is less than 0.6% when the mesh number is increased from 361,345 to 433,680. Therefore, the mesh number was set to not less than 361,345 in the simulation.

## 4. Results and Discussion

### 4.1. Distribution of Concentration Field in the Microchannels

**Figure 2:**
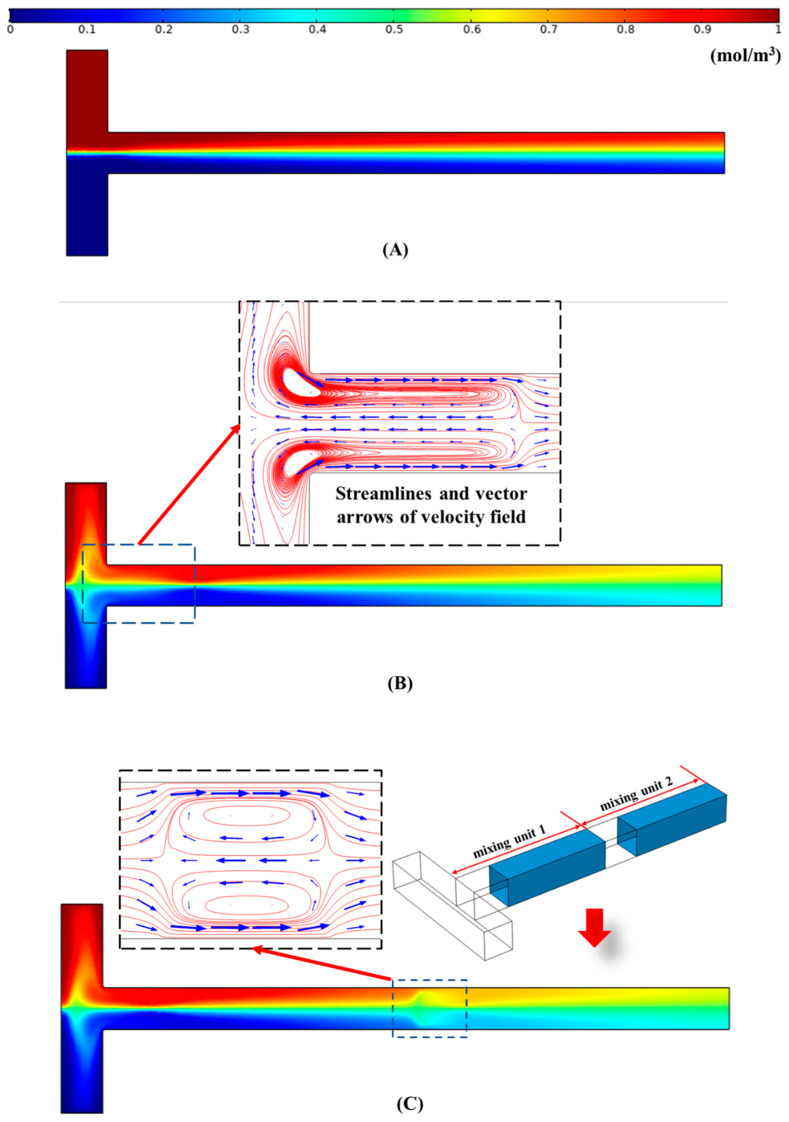
Distribution of the concentration field on the center cut plane of the microchannel under different cases: (**A**) The channel wall has uniform zeta potential (−50 mV); (**B**) the channel wall has non-uniform zeta potential (one mixing unit: *ζ*_u_ = −50 mV, *α* = 0.1, *β* = 6); (**C**) the channel wall has non-uniform zeta potential (two mixing units: *ζ*_u_ = −50 mV, *α* = 0.1, *β* = 6).

Figure 2 shows the distribution of the concentration field in the microchannels in steady-state under different conditions. Assuming that all the walls are unmodified, namely, all the walls have the same zeta potential, the concentration field distribution in the microchannels is shown in Figure 2A. It is obvious that the mixing effect of the solute is poor in this case. By contrast, when the downstream of the outlet is designed with a smaller absolute value of zeta potential (see Figure 1), the distribution of the concentration field is shown in Figure 2B (The zeta potential ratio and the length ratio of the modified section to the unmodified section were set to be *α* = 0.1 and *β* = 6, respectively). It can be easily seen that the mixing performance, in this case, is much better than that in Figure 2A. The main reason is the generation of the electrokinetic vortices due to the “impede effect” of the low flow velocity liquid in the modified section to the high flow velocity liquid in the unmodified section, as displayed in the inset of Figure 2B. The electrokinetic vortices enhance the convection and diffusion effect and the corresponding mixing performance.

The modified section and unmodified section of the outlet channel can be seen as a mixing unit. There is one mixing unit in Figure 2B. If two mixing units were set in the outlet channel, what would happen? As shown in Figure 2C, two same size mixing units were set. The length of the outlet channel, the zeta potential ratio, and the length ratio of the two mixing units in Figure 2C are the same as that in Figure 2B. It is obvious that extra vortices form in the unmodified section of the mixing unit 2, as displayed in the insets of Figure 2C. Therefore, the mixing performance in Figure 2C is better than that in Figure 2B. However, since the extra vortices in Figure 2C are weak, the improvement of the mixing effect is limited.

Besides, to evaluate the presented micromixer, a similar micromixer reported by other researchers is compared. Biddiss et al. [42] designed a similar micromixer to the micromixer presented in this paper and investigated the liquid mixing performance in the microchannel. They set a complex patch patterning on the walls of a T-type microchannel and the patch patterning have different value and different polarity zeta potentials. Their study shows that for a 95% mixture, the required mixing length of the outlet channel is more than 2 mm under the conditions of the diffusion coefficient 4.37 × 10^−10^ m^2^/s and the electric field 280 V/cm (The Reynolds number is about 0.35.). To evaluate the introduced micromixer, the required mixing length for a 95% mixture is also calculated under the same diffusion coefficient and Reynolds number. The numerical result shows that the required mixing length of the introduced micromixer for a 95% mixture is less than 1 mm. The micromixer reported by Biddiss et al. needs to set the channel walls with a complex patch patterning via surface modification technology. By contrast, for the micromixer presented in the paper, only the downstream part of the outlet channel needs to be treated. Thus, the fabrication of the introduced micromixer is relatively easier.

### 4.2. Effect of Zeta Potential Ratio (α) on Mixing Index

**Figure 3:**
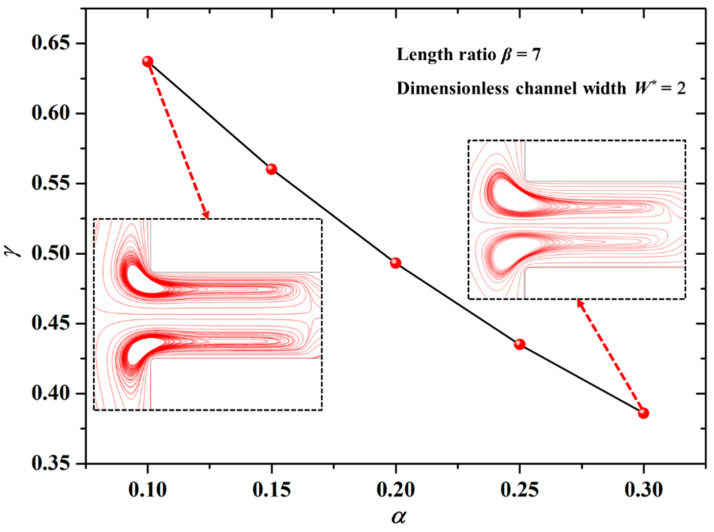
Dependence of the zeta potential ratio on the mixing index under the conditions of the length ratio *β* = 7 and the dimensionless channel width W* = 2 (W* = *W*/*L*_ref_, *L*_ref_ = 10 μm).

The dependence of the zeta potential ratio (*α*) on the mixing index (*γ*) is shown in Figure 3 under the conditions of the length ratio *β* = 7 and the dimensionless channel width W* = 2 (W* = *W*/*L*_ref_, *L*_ref_ = 10 μm). It is clear in Figure 3 that the mixing index (*γ*) of the solute declines with the increase of the zeta potential ratio (*α*) of the modified section to the unmodified section. It could be explained as follows:

The larger value of α means the absolute value difference of the zeta potentials between the unmodified section and the modified section is smaller, accordingly, the velocity difference of EOFs formed in the two sections is smaller. As a result, the “impede effect” of the low flow velocity liquid in the modified section to the high flow velocity liquid in the unmodified section is weaker, hence, the electrokinetic vortices formed in the channel are weak, as shown in the insets of Figure 3. Therefore, it is not difficult to understand the changing trend of *γ* with *α* in Figure 3.

### 4.3. Effect of Length Ratio (β) on Mixing Index

**Figure 4:**
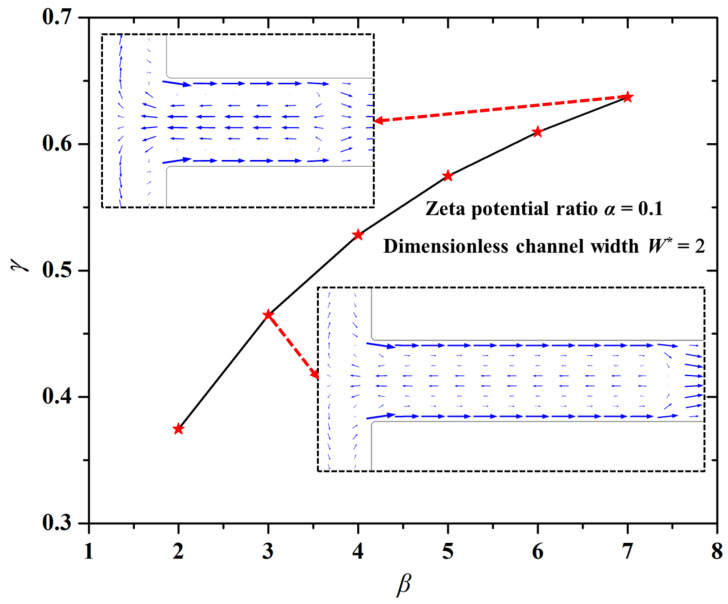
Dependence of the length ratio on the mixing index. The other parameters were set to be the zeta potential ratio *α* = 0.1 and the dimensionless channel width W* = 2.

Figure 4 shows the changing trend of the mixing index (*γ*) with the length ratio (*β*) of the modified section to the unmodified section. The other parameters were set to be the zeta potential ratio *α* = 0.1 and the dimensionless channel width W* = 2. One can easily see in Figure 4 that the larger the value *β*, the higher the mixing index (*γ*). Namely, the mixing performance is better under the larger length ratio (*β*) of the modified section to the unmodified section.

As mentioned above, the liquid in the modified section with a smaller EOF velocity will impede the flow of liquid in the unmodified section with a higher EOF velocity. The larger value of *β* signifies the length difference between the modified section and the unmodified section is larger. As a result, the “impede effect” of modified section liquid to the liquid in the unmodified section will become stronger, hence, the rotation velocity of vortices formed in the channel will be higher, as displayed in the insets of Figure 4. Thus, the mixing performance becomes better with the increase of the length ratio (*β*).

### 4.4. Effect of Channel Width (W) on Mixing Index

**Figure 5:**
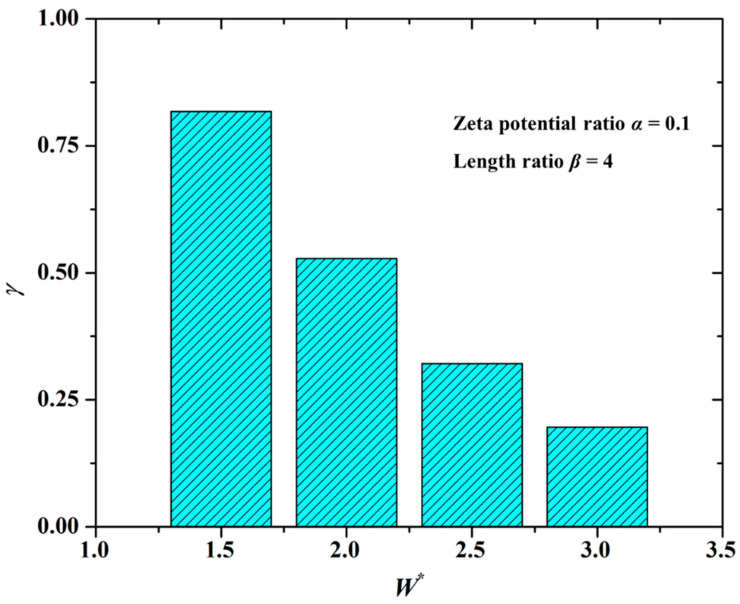
Dependence of the channel width on the mixing index under the conditions of the zeta potential ratio *α* = 0.1 and the length ratio *β* = 4.

The channel width greatly affects the mixing performance of the solute, as shown in Figure 5 under the conditions of the zeta potential ratio *α* = 0.1 and the length ratio *β* = 4. From Figure 5 it is found that the mixing index (*γ*) is smaller under the larger dimensionless value of the channel width (W*). In other words, the mixing performance is better in the microchannel with a smaller channel width. The explanation is as follows.

The “impede effect” of the modified section liquid to the unmodified section liquid determines the formation of electrokinetic vortices and hence the mixing performance. Because the velocity of EOF formed on the walls of the modified section is low, with the decrease of the channel width, the channel walls will squeeze the liquid, thereby enhancing the “impede effect” of the modified section liquid. Assuming that the channel width is quite large, the above-mentioned “squeeze effect” from the modified section walls will become quite weak. As a result, the rotation velocity of the vortices is very low, even the vortices disappear. Therefore, the mixing index (*γ*) decreases with the increase of the channel width, as shown in Figure 5.

## 5. Conclusions

Liquid mixing based on the electrokinetic vortices generated in a T-type microchannel with nonuniform but same polarity zeta potentials is numerically investigated in this work. The outlet channel of the T-type microchannel is composed of a modified section with a smaller zeta potential and an unmodified section with a larger zeta potential. The results show that the “impede effect” of the modified section liquid to the unmodified section liquid determines the formation of electrokinetic vortices and hence the mixing performance. The larger the EOF velocity difference of the unmodified section to the modified section is, the stronger the “impede effect” is. The “impede effect” can be enhanced by decreasing the channel width and the zeta potential ratio of the modified section to the unmodified section (namely, increasing the zeta potential difference of the unmodified section to the modified section) and increasing the length ratio of the modified section to the unmodified section, thereby improving the mixing performance.

The work provides a basic understanding of liquid mixing in a T-type microchannel with nonuniform but same polarity zeta potentials. The presented micromixer is quite simple in structure and can be fabricated easily. Since the micromixer only needs a small battery to work, the work system of this micromixer is also simple. Therefore, the above advantages of this micromixer are conducive to the integration and miniaturization of microfluidic devices.
